# Vectorized dataset of silted land formed by check dams on the Chinese Loess Plateau

**DOI:** 10.1038/s41597-024-03198-z

**Published:** 2024-04-06

**Authors:** Yi Zeng, Tongge Jing, Baodong Xu, Xiankun Yang, Jinshi Jian, Renjie Zong, Bing Wang, Wei Dai, Lei Deng, Nufang Fang, Zhihua Shi

**Affiliations:** 1 State Key Laboratory of Soil Erosion and Dryland Farming on the Loess Plateau, Institute of Soil and Water Conservation, Northwest A&F University, 26 Xinong Road, Yangling, Shaanxi Province 712100 P. R. China; 2 Institute of Soil and Water Conservation, Chinese Academy of Sciences and Ministry of Water Resources, 26 Xinong Road, Yangling, Shaanxi Province 712100 P. R. China; 3 College of Resources and Environment, Huazhong Agricultural University, Wuhan, 430070 P. R. China; 4 School of Geographical Sciences, Guangzhou University, Guangzhou, 510006 P. R. China

**Keywords:** Geomorphology, Hydrology

## Abstract

Check dams on the Chinese Loess Plateau (CLP) have captured billions of tons of eroded sediment, substantially reducing sediment load in the Yellow River. However, uncertainties persist regarding the precise sediment capture and the role of these dams in Yellow River flow and sediment dynamics due to the lack of available spatial distribution datasets. We produced the first vectorized dataset of silted land formed by check dams on the CLP, combining high-resolution and easily accessible Google Earth images with object-based classification methods. The accuracy of the dataset was verified by 1947 collected test samples, and the producer’s accuracy and user’s accuracy of the dam lands were 88.9% and 99.5%, respectively. Our dataset not only provides fundamental information for accurately assessing the ecosystem service functions of check dams, but also helps to interpret current changes in sediment delivery of the Yellow River and plan future soil and water conservation projects.

## Background & Summary

**Fig. 1:**
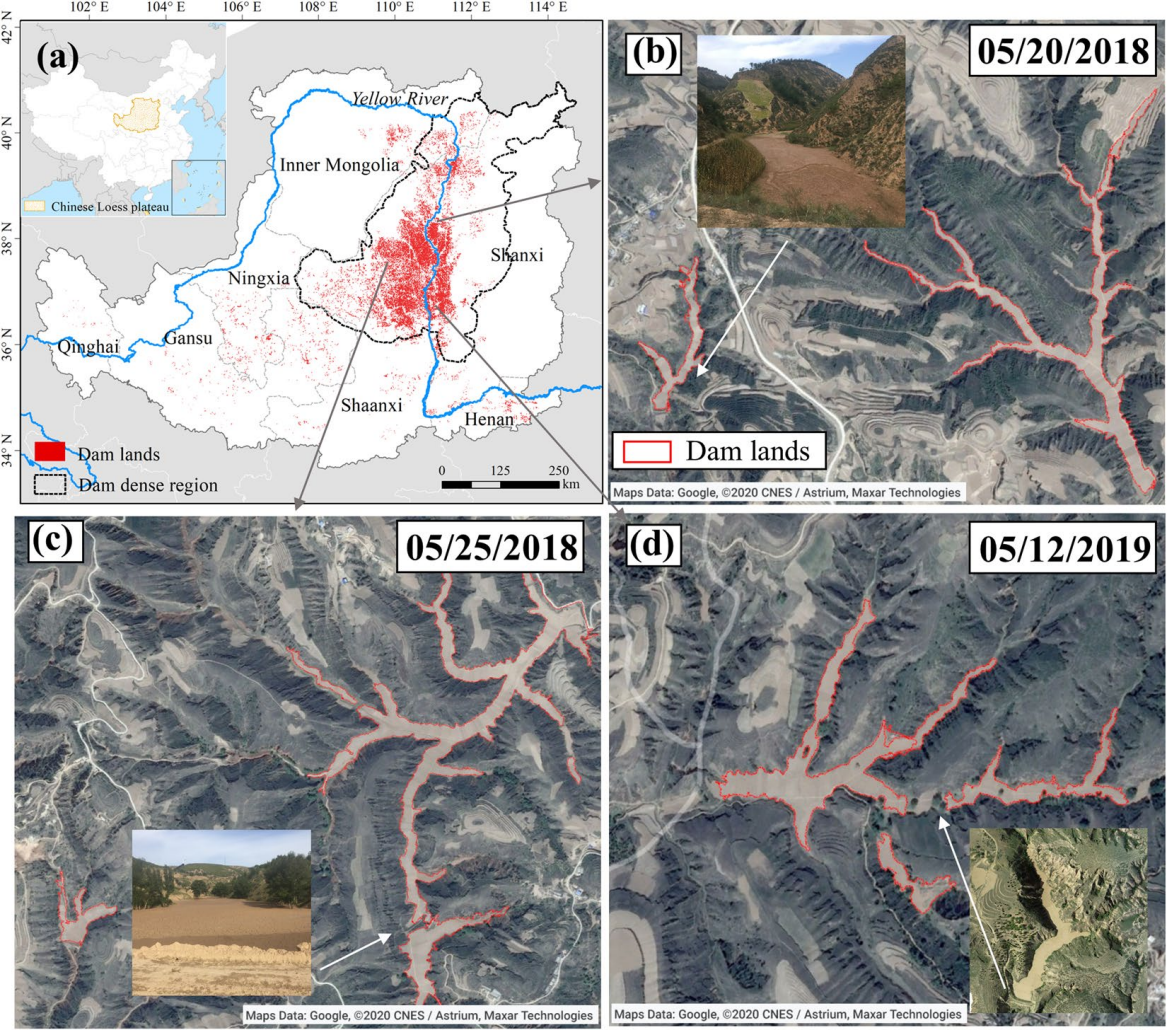
Check dams and dam lands on the Chinese Loess Plateau. (**a**) Dam concentration region on the Chinese Loess Plateau, (**b**–**d**) Google Earth images, photographic images, and unmanned aerial vehicle images of dam lands in May.

Accelerated soil erosion has caused worldwide land degradation, water pollution, and grain yield reduction, which has become one of the most pressing environmental issues threatening human sustainable development^1,2^. In order to alleviate severe soil erosion, many countries have implemented a series of soil and water conservation projects according to local conditions in recent years, including vegetation restoration, conservation tillage^3^, terrace and check dam construction^3–5^. Among them, check dams have played a significant role in soil and water conservation and are widely applied in global arid and semi-arid regions with severe soil erosion^6,7^. The check dams built in the gullies can directly intercept the eroded sediment from the watershed^8^. Additionally, the silted land behind the check dam formed by the eroded sediment (hereinafter referred to as dam land) (Fig. 1), can reduce the gully slope and surface runoff velocity, stabilizes the gully bed, and weakens erosive energy^9^. The “sediment retaining” and “erosion reduction” of the check dams and dam lands synergistically reduce the watershed sediment yield. According to results at different study areas such as China, Spain, and America, check dams and dam lands can reduce sediment yield by about 50–77% at the watershed scale^10–12^. In addition to preventing soil erosion, the construction of check dams and the subsequent formation of dam lands also provides more additional ecosystem services, including carbon sequestration and grain supply. Large amounts of organic-rich eroded sediments are buried and effectively preserved in dam lands, becoming an important terrestrial carbon sink^13^. Moreover, fertile dam lands have high water content and strong drought resistance, is often used as cropland with high and stable grain production^14^.

The Chinese Loess Plateau (CLP) is the region with the largest number and the densest distribution of check dams in the world due to its severe soil erosion in the past few decades^15^. Since the 1970s, the Chinese government and local farmers have widely built check dams in the gullies to prevent sediment loss to the Yellow River, which used to have the largest riverine sediment flux in the world. Tens of thousands of check dams, cooperating with other soil and water conservation measures such as vegetation restoration and terraces, have greatly reduced the sediment load into the Yellow River from the CLP by ~85% in recent decades (from ~1.6 Pg yr^−1^ in the period 1919–1960 to ~0.25 Pg yr^−1^ in the period 2010–2016)^16^. However, there is still a large uncertainty about how much sediment has been intercepted by check dams and what roles they play in the flow and sediment variations in the Yellow River because the number and spatial distribution of check dams and dam lands are still unclear^17^. The Ministry of Water Resource of the People’s Republic of China reported that more than 110,000 check dams had been constructed, and they captured about 21 × 10^9^ m^3^ sediment on the CLP^18^. Yet, the Bulletin of First National Census for Water reported only 58,446 check dams had been constructed and remained intact till 2013 and the sediment volume is about 7 × 10^9^ m^3^ (ref. ^19^). Almost all regional studies regarding the sediment budget of the Yellow River referred to one of these two reports, and the huge discrepancy between the check dam amounts in these reports results in the possibility that all sediment retention and carbon storage estimated using these data may be overestimated or underestimated^16,20,21^. Noticeable, solely determining the spatial information of check dams is insufficient to ascertain their sediment retention capacity. In contrast, the dam lands formed by check dams epitomizes the concentrated manifestation of check dams’ sediment retention function, and accurately identifying them is an important prerequisite for determining the amount of sediment trapped by the check dams. Moreover, once the spatial information of the dam lands are acquired, it becomes considerably easier to locate check dams near the dam lands^22^. Therefore, it is necessary to generate the spatial distribution of dam lands on the CLP, which is an important prerequisite to accurately quantify the contribution of check dams on the CLP to the variation of sediment load in the Yellow River and to evaluate the carbon sequestration and grain supply benefitting from check dams.

The early check dams and dam lands data mainly comes from the field survey organized by government departments, which may lead to some statistical errors at the regional scale^23^. In recent decades, remote sensing has been widely used to obtain regional, national, and even global land cover or land use, and has made great progress in fields of cropland, terraces, water identification, and so on^24–26^. However, currently, only a few studies have explored the possibility of obtaining dam lands information based on remote sensing technology^27,28^. Tian *et al*.^28^ obtained the spatial distribution of dam lands in the Huangfuchuan watershed based on the supervised classification and Landsat-5 TM images. Li *et al*.^27^ proposed a method integrating deep learning and object-based classification to identify dam lands within a range of tens to hundreds of km^2^. Nevertheless, these studies have only explored different methods to identify dam lands on a very small scale, and have not be extended to the whole CLP. That is, the current dataset of dam lands on the CLP is still lacking.

Several key difficulties limit the current identification of dam lands at regional scale. Firstly, check dams on the CLP are mainly small and medium-sized check dams, with a correspondingly dam land area of 0.2–2 hm^2^ (ref. ^29^). The decametric-resolution images (e.g., Landsat-7/8) may generate erroneous judgement for identification of dam lands due to the limited number of pixels and jagged edges^30^. Secondly, the dam lands are usually used for planting crops^31^, which are difficult to distinguished from the surrounding slope croplands and terraces in terms of spectral characteristics. Finally, dam lands have the characteristics of large spatial heterogeneity and patch fragmentation, which are more difficult to be identified than other land use types^27^. The traditional pixel-based identified methods usually focus on medium resolution images, seldom refer to the structure and texture characteristics and the correlative information between adjacent pixels^32,33^. Meanwhile, these methods may lead to salt-and-pepper noise, which reduces the accuracy of object classification^34^. In contrast, the object-based classification method comprehensively considers a series of factors, such as spectral features, shape, size, texture, and adjacency, can obtain high-precision identified results combing with high spatial resolution images^35^. Therefore, we attempt to identify dam lands on the CLP using object-based classification method in conjunction with high spatial resolution (0.3–1.0 m) and easily accessible Google Earth images in this study. The self-development computer program in conjunction with auxiliary data, visual interpretation and expert knowledge are used to improve the identified accuracy of dam lands. The accuracy of this dataset is verified by the validation samples obtained through field surveys and Google Earth. This study provides basic data for researchers to quantify the ecosystem service of check dams, and offers useful information for policy makers to plan soil and water conservation projects.

## Methods

**Fig. 2:**
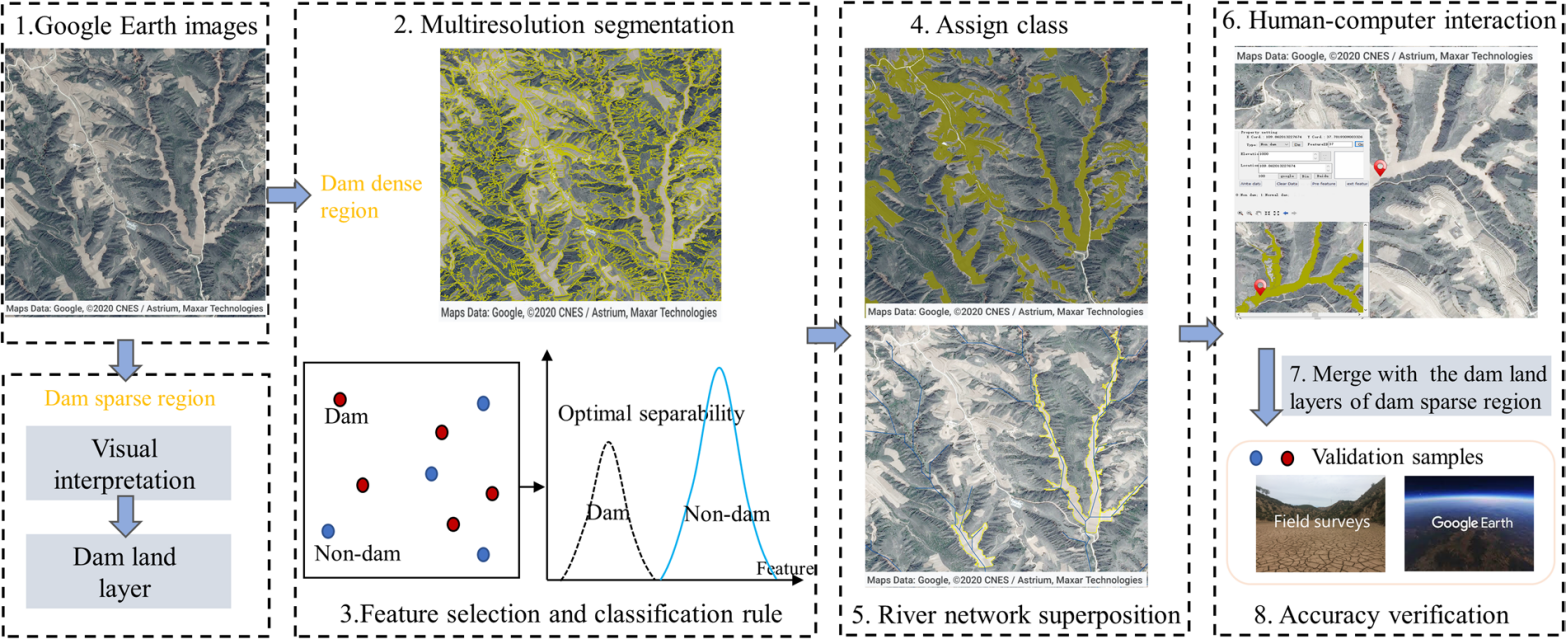
The workflow of dam lands identification on the Chinese Loess Plateau.

According to previous reports, the hilly and gully region occupies 33% (2.1 × 10^5^ km^2^) of the area of the CLP (6.4 × 10^5^ km^2^), but about 85% of the check dams are distributed here^17^. Therefore, to improve the identified efficiency of dam lands, we divided the CLP into dam dense region (hilly and gully region) and dam sparse region (other region) according to the spatial distribution characteristics of check dams. For the dam dense region, we identified dam lands mainly through high-resolution Google Earth images and object-based classification method (Fig. 2). However, owing to the large area but with just about 15% of the check dams in the dam sparse region, the acquisition and processing of corresponding Google Earth images is time-consuming. Therefore, we identified the dam lands in dam sparse region by artificial visual interpretation in Google Earth. We then aggregated the dam lands layers of these two regions and verified the accuracy with the validation samples obtained through field surveys and Google Earth.

### Satellite images collection

**Fig. 3:**
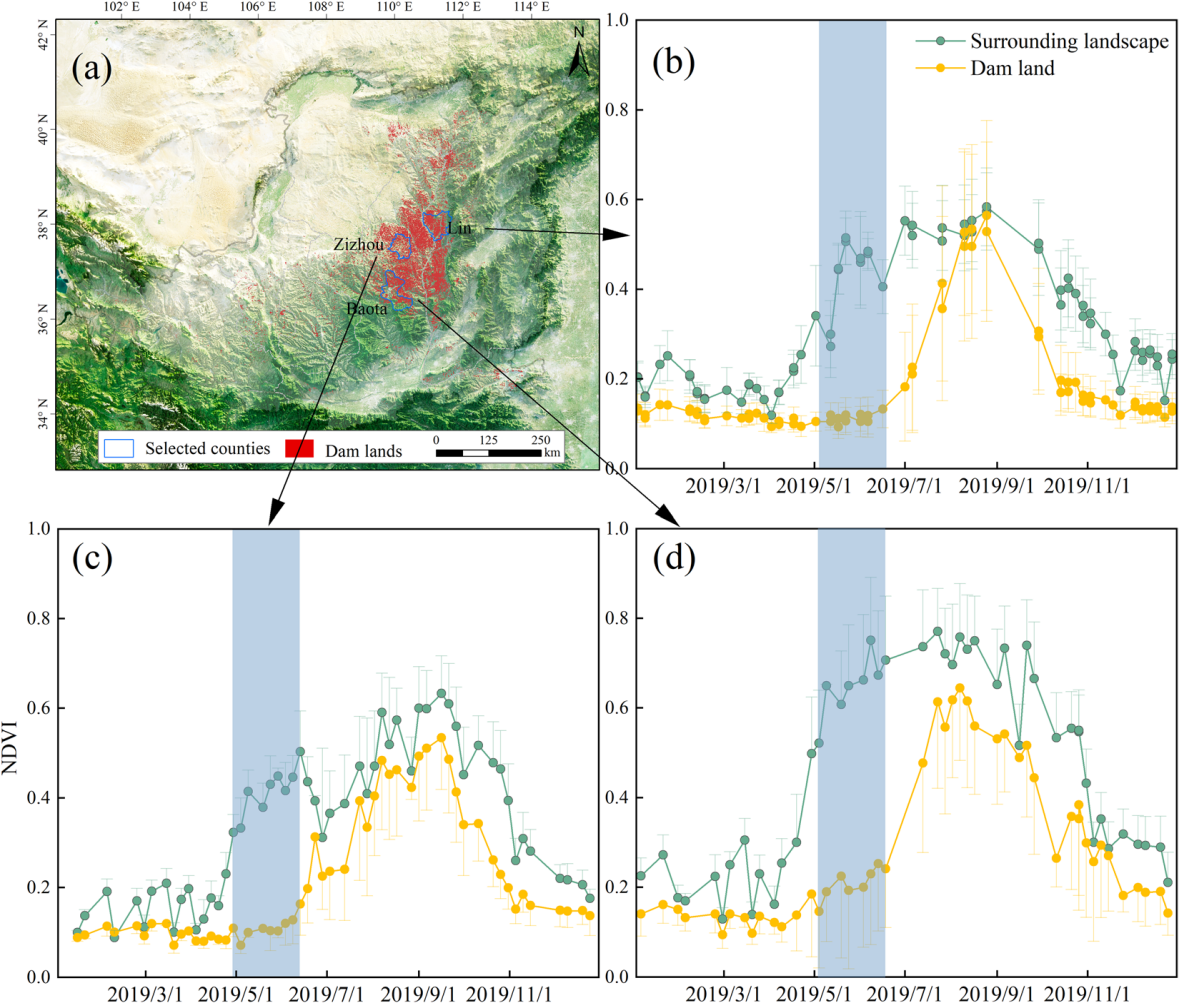
(**a**) Location of the three selected counties, (**b**–**d**) NDVI time series of the dam lands and surrounding landscape of the three selected counties. The blue rectangle represents the period with the largest NDVI difference between the dam lands and the surrounding landscape.

Dam land is one of the most productive land types on the CLP, so the majority of it is cultivated. Therefore, the identification object in this study is the cultivated dam lands on the CLP, excluding water-covered dams and abandoned dam lands. Farmers living on the CLP usually plough the dam lands around May each year^36^, while the gullies around the dam lands are covered with vegetation, which makes it easy to distinguish the dam lands from the surrounding landscape on satellite images. The distinguishable difference can be confirmed by the field investigations (Fig. 1). Additionally, we randomly selected dam lands (N_dam=_127) and surrounding landscape samples (N_sur_ = 130) in three counties with a dense distribution of check dams (Baota county: N_dam_ = 41 and N_sur_ = 48; Zizhou county: N_dam_ = 37 and N_sur_ = 45; Lin county: N_dam_ = 49 and N_sur_ = 37). Then, combined with the Sentinel-2 image, the NDVI time series values of these sample points in 2019 were calculated in Google Earth Engine. We also found that the most significant difference in NDVI between the dam lands and the surrounding landscape around May 2019 (Fig. 3b–d). Therefore, we collected all available images from May 2016 to 2020 in the dam dense region in Google Earth, with spatial resolution of 0.3–1.0 m. It is worth noting that the images in Google Earth are usually spliced from images retrieved in different periods or from different sensors, which may result in potential chromatic aberration at the junction of two image scenes^37^. To avoid subsequent segmentation errors caused by image chromatic aberration, we divided images of the dam dense region according to the shooting date and obtained a total of 52 images.

### Multiresolution segmentation

**Fig. 4:**
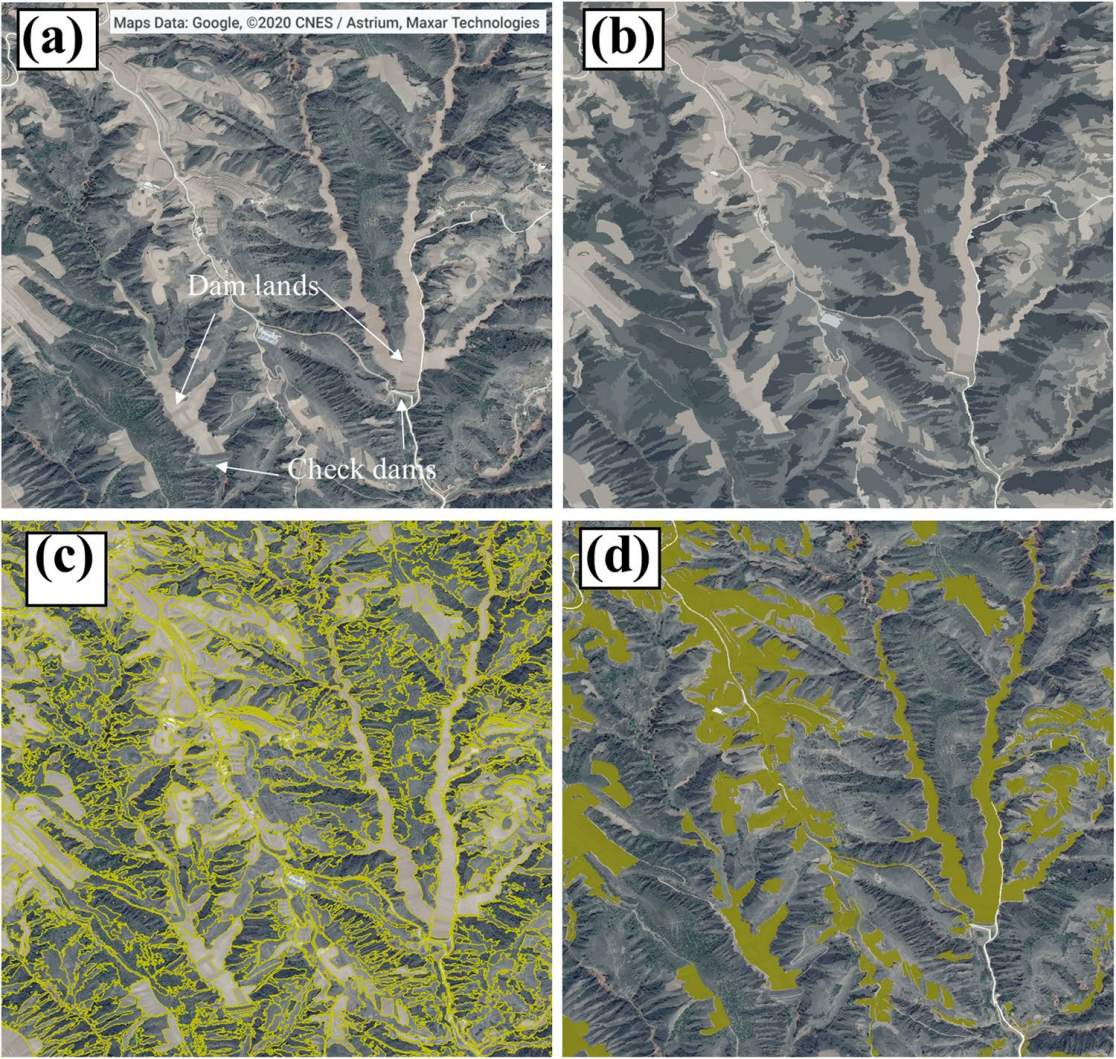
Multiresolution segmentation of dam lands. (**a**) Original Google Earth image; (**b**) Object mean view in eCognition Developer; (**c**) View after multiresolution segmentation; (**d**) Classify the segmented objects. The yellow polygon is the identified target category.

Here we used multiresolution segmentation algorithm in the eCognition Developer 9.0 (Trimble, Sunnyvale, California, USA). This segmentation method achieves image segmentation by merging adjacent pixels with similar features under the premise of ensuring the maximum homogeneity between the pixels within the object^38^. The scale, shape, and compactness are the most important parameters in multiresolution segmentation algorithms, and their values will affect the segmentation results^39^. The scale parameter is used to determine the maximum heterogeneity of the generated object and to control the size of the segmented object. The estimation of scale parameter 2 (ESP2) plugin in eCognition Developer can automatically evaluate the segmentation effect based on the local variance (LV) and its rate of change (ROC). Therefore, we used the ESP2 plugin to determine the optimal scale parameter for dam lands identification^40^. Settings of shape and compactness is also crucial for the segmentation and subsequent identification of dam lands, as the dam lands are usually narrow and irregular in shape. Therefore, we made different combinations of these parameters to test the best parameter settings in combination with visual inspection. Finally, we set the scale parameter, shape weight, and compactness weight to 100, 0.7, and 0.3 to obtain the best segmentation results (Fig. 4).

### Feature selection and identification rule

Here we used the assigned class algorithm in the eCognition Developer, which determines the category of segmented objects by setting threshold. When there were significant differences between the target and background categories for some features, the assigned class algorithm could be used to construct identification rules^41^. The assigned class algorithm was more suitable for our study than other identification methods, as our goal is to identify more dam lands in a target category. For each segmented image, we firstly randomly selected 30–80 dam lands and non-dam lands at different locations according to the amplitude of the image. Then, we used the separability and thresholds algorithm in eCognition Developer to automatically select identification features (e.g., red, green, and blue band, shape, texture, and brightness) and determine the threshold of selected feature^42^ (Fig. 1). Finally, we used the feature threshold to classify the segmented objects and manually adjusted the threshold range using visual interpretation to ensure that all dam lands were included in the threshold range. Through the above steps, we obtained the bare land layer in the dam dense region in May, mainly including the dam lands in gullies and the cropland on slope land (Fig. 4).

### River network superposition and human-computer interaction

**Fig. 5:**
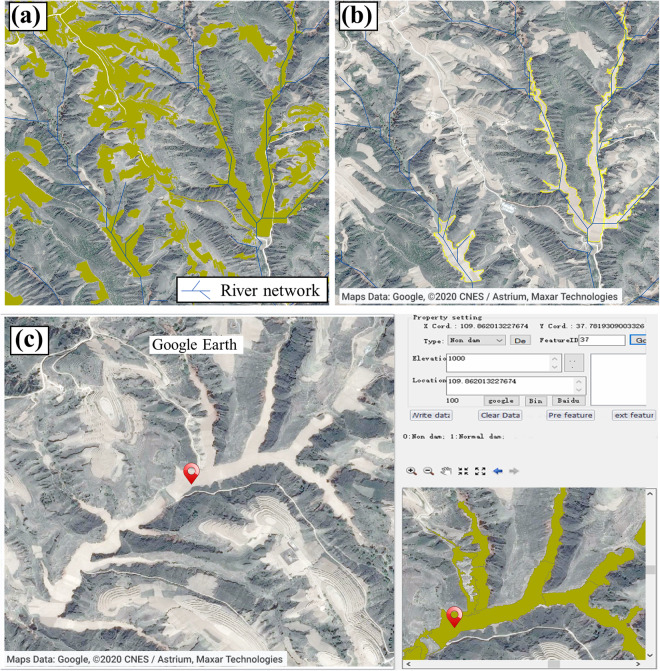
(**a**) Classified objects superimposed on river network, (**b**) removal of non-dam lands by superimposed river network; (**c**) human-computer interaction.

All dam lands on the CLP are distributed in gullies, which can be easily separated from non-dam lands on the slope through the river network. We first used Shuttle Radar Topography Mission digital elevation model (SRTM-DEM) with a spatial resolution of 30 m to extract river networks in ArcGIS 10.6 (Esri, Southern California, USA). Then we overlaid the river network layer on the bare land layer to identify the dam lands in the gullies (Fig. 5a,b). In order to enhance the identified accuracy of dam lands, we developed a convenient human-computer interaction program to mark non-dam land vector polygons, such as some natural floodplains and slope cropland that were captured by superimposed river networks. The left window, displaying a high-resolution Google Earth image, can be linked to the right window, which represents the potential dam lands layer identified in the previous steps (Fig. 5c). We assigned values to each identified vector polygon (e.g., non-dam land is 0, dam land is 1) based on auxiliary data, visual interpretation, and expert knowledge. Finally, we merged the vector polygons identified from 52 images with the attribute value of 1 in ArcGIS, which is the final dam lands layer in the dam concentration region.

### Estimation of sediment volume of check dam

Our recent research has established a method for estimating the sediment volume of check dams based on unmanned aerial vehicle (UAV) photogrammetry and proposed an area-volume empirical formula for estimating the volume of check dams at regional scale^15^. Based on this area-volume empirical formula, we can accurately estimate the sediment volume of check dams according to the area of each dam land. A more detailed description and accuracy verification of this sediment volume estimation for check dams can be found in Zeng *et al*.^15^.

1 $$V=\left\{\begin{array}{ll}\left(6.31\pm 0.03\right){D}_{area}^{(1.33\pm 0.01)} & \left(0\le {D}_{area} < 2\right)\\ \left(3.80\pm 0.07\right){D}_{area}^{(1.51\pm 0.01)} & \left(2\le {D}_{area} < 5\right)\\ \left(8.48\pm 0.89\right){D}_{area}^{(0.96\pm 0.02)} & \left(5\le {D}_{area}\right)\end{array}\right\}$$

where V is the sediment volume of check dam (×10^4^ m^3^); D_area_ is the area of dam land (hm^2^).

## Data Records

The generated dam lands dataset in this article is freely available at 10.5281/zenodo.7857443^43^. The vectorized dataset of dam lands is formatted as a shapefile (.shp), where each record is depicted as a polygon and encompassing the subsequent attributes: longitude, latitude, dam land area, dam land perimeter, sediment volume, and sediment mass. In the attributes table, the field *Area* (unit: m^2^) and *Shape_Leng* (unit: m) represent the area and perimeter of the dam land polygon, calculated in WGS_1984_EASE_Grid_Global coordinates; the field *volume* (unit: m^2^) and *mass* (unit: m) represent the sediment retention volume and mass of check dams.

## Technical Validation

At present, there is no available spatial distribution dataset of dam lands on the CLP. Therefore, we took the test samples obtained through field surveys and Google Earth to verify the identification accuracy of dam lands in our dataset. From 2017 to 2023, we conducted many field surveys on the CLP and recorded the detailed spatial information of 102 dam lands. Then we obtained more available validation samples through visual interpretation on Google Earth. Due to the non-uniform spatial distribution of dam lands, the traditionally uniform sampling may lead to the deviation of accuracy evaluation. To improve the reliability of verification, we determined the number of test samples in each county according to the number of dam lands in our dataset at the county level. That is, we allocated more test samples in counties where check dams are dense. A total of 1947 test samples were acquired within the study area, of which 949 samples were interpreted as dam lands and 998 samples as non-dam lands.

We evaluated the accuracy of dam lands by calculating the confusion matrix, including producer’s accuracy (PA), user’s accuracy (UA), and Kappa coefficient. The results of the confusion matrix show that the overall accuracy (OA) of the dam lands is 94.4%, and the PA and UA of the dam lands are 88.9% and 99.5%, respectively. The non-dam lands had both UA and PA over 90%. Additionally, the kappa coefficient is 0.89, indicating that the dataset has high classification accuracy (Table 1).

**Table 1:** Accuracy evaluation of dam lands with 1947 random test samples.

Class	Non-dam land	Dam land	Producer’s accuracy (%)	User’s accuracy (%)
Non-dam land	994	105	99.6	90.4
Dam land	4	844	88.9	99.5
Overall accuracy = 94.4%			Kappa = 0.89	

It is worth noting that we have used an identification method that seems to be outdated and time-consuming to produce the first dataset of dam lands formed by check dams on the CLP, but this is currently the only method that can accurately extract dam lands across the CLP. The CLP region spans an expansive 640,000 km^2^ and can be divided into six different geological divisions^15^. Significantly, each geological division exhibits pronounced variations in the dimensions, morphology, and spectral characteristics of dam lands, which poses a huge challenge to objective machine-learning or other automated detection schemes. While there exists a few deep learning methodologies for the extraction of dam lands^27,44^, their applicability is typically constrained to a watershed scale. Furthermore, in comparison to the dam land contours extracted in these prior studies, our proposed workflow demonstrates superior identification performance (Fig. 7). Since this is the first vectorized dataset of dam lands within the CLP, achieving a broader spatial coverage and enhanced identification performance assumes particular significance. We believe that our dataset will contribute to the future continuous efforts in automating, which is part of a larger effort to automatically identify such land use.

### Uncertainty, limitation, and future work

This study provides the spatial distribution dataset of dam lands at the regional scale on the CLP for the first time. The object-based classification method used in this study demonstrated high accuracy and good visual effect, especially in combination with the self-development computer program. Moreover, besides providing spatial distribution information, we also offer data on sediment volume and sediment retention capacity of the check dam. This information is crucial, yet currently lacking, and it can serve as valuable references for regional soil and water conservation planning. However, there are still some limitations in the identification of dam lands on regional scale. Firstly, to ensure accuracy of the basic dataset provided for the first time, the whole identification process is still semi-automatic, and visual interpretation and expert experience are still needed in the steps of identification feature selection and human-computer interaction. Secondly, there are a few water-covered check dams on the CLP, and their characteristics and functions are same as small reservoirs. It is therefore difficult to distinguish water-covered check dams from reservoirs, which may lead to a slight underestimation of the number of dam lands in our dataset. Finally, due to the randomness and accessibility of historical Google Earth images, it is difficult to obtain the images for the entire CLP in April and May of the same year. Considering that the large-scale ecological restoration projects (e.g., Grain for Green project) on the CLP have significantly reduced soil erosion, the change of dam land area in the short term is basically negligible. Therefore, we collected all available images covering most study areas in May from 2016 to 2020. However, some newly constructed check dams may have been ignored (e.g., the check dam was constructed in 2018, but it was not imaged in 2016), which may also lead to a slight underestimation of the number of dam lands. In future work, our dataset can be utilized as the training set, in conjunction with high-resolution satellites equipped with additional multispectral bands (e.g., Sentinel-2) and deep learning techniques, to propose an enhanced, automated, and convenient process for dam lands identification.

## Usage Notes

In recent years, check dams on the CLP have been paid more and more attention by researchers because of their important roles in soil and water conservation. Using “check dam” and “Loess Plateau” as keywords, 8450 research articles were searched on Google Scholar, among which 3540 were published after 2018. To date, most studies have relied on time-consuming and laboriously field investigations to find suitable check dams satisfied with the research objectives^45,46^. Our dataset can provide the precisely spatial location for check dams and dam lands, which greatly saves the manpower and material resources of researchers. More importantly, our dataset also offers key parameters such as dam land area and sediment volume of check dams for those studies which use check dams to calculate soil erosion rate^47^, estimate watershed sediment delivery ratio^8^, and validate the soil erosion model^12^.

**Fig. 6:**
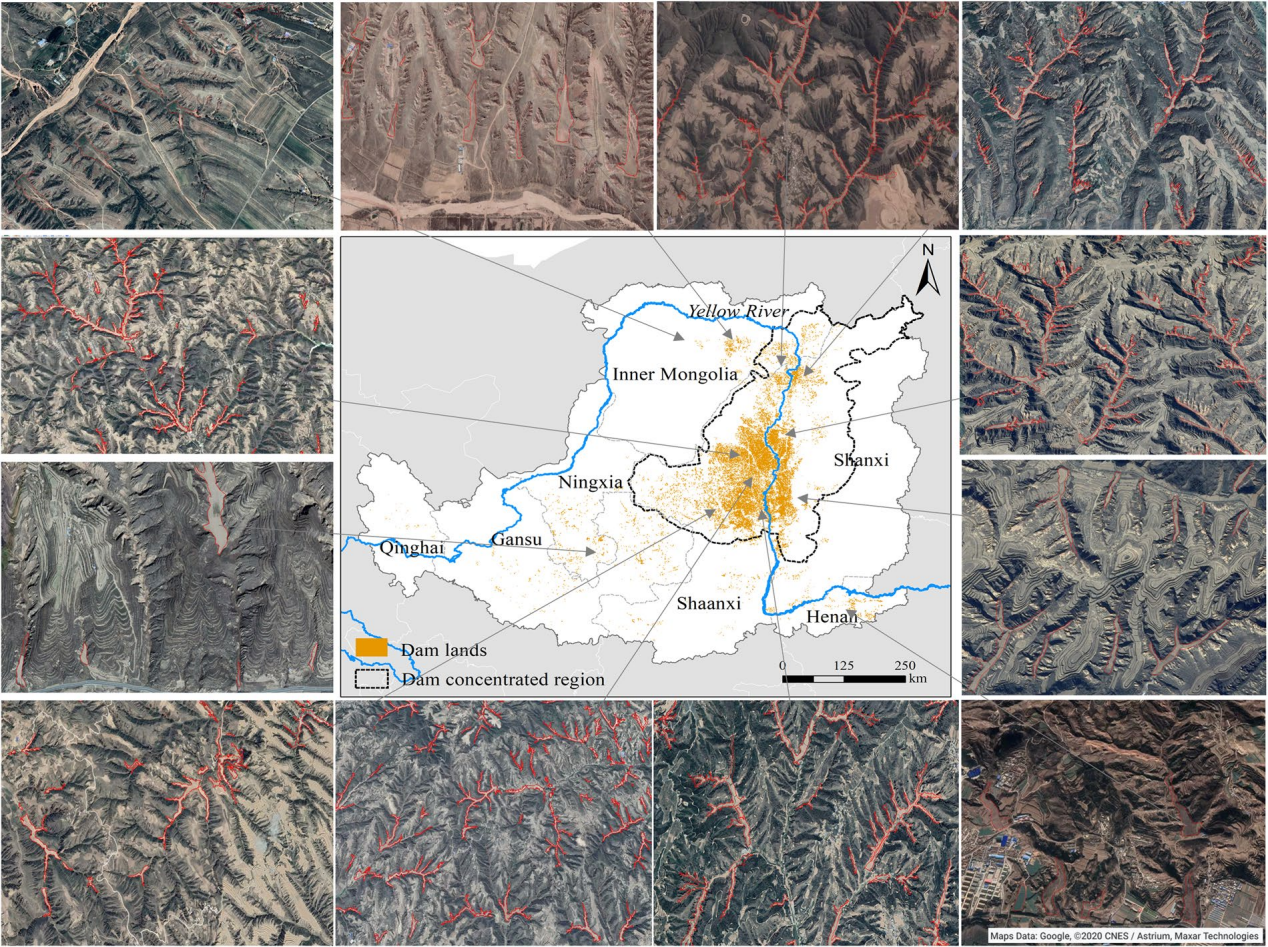
Spatial distribution of check dams on the Chinese Loess Plateau.

For policymakers, our database will provide detailed data on the spatial distribution (Fig. 6), dam land area, and sediment volume of check dams on the CLP. When these key parameters of check dams are obtained, we can conveniently and accurately evaluate the ecosystem services functions of check dams, including sediment retention^48^, carbon sequestration^49^, and grain supply^50^. We preliminarily estimated that check dams on the CLP have intercepted about 10.2 × 10^9^ tons eroded sediment during 1970–2020, equalling 46% of the sediment load of the Yellow River, which was once the largest sediment contributor to the global ocean. These data will be beneficial to understand the anthropogenic influences on the enormous changes of sediment in the Yellow River^16^. Additionally, recent review article emphasizes the carbon sequestration potentiality of check dams, and points out that there are still great uncertainties and challenges in the estimation of carbon storage of check dams on regional scale^13^. When the sediment carbon content of check dams is obtained by combining macro-scale filed sampling with meta-analysis, the carbon storage of check dams can be easily estimated by using our dataset^51^. Moreover, the soil silted by the check dams has higher moisture than that of terrace and slope cropland. Previous study showed the grain yield of dam lands is 2~3 times that of terraced fields and 6~10 times that of slope cropland^14,50^. Our dataset can be used to estimate the grain supply of check dams, combined with the grain yield per unit area of dam lands. Finally, according to the Chinese government’s plan, 56,161 new check dams will be built on the CLP by 2030^52^. Our spatial distribution data of check dams and dam lands provides important information for optimizing the location of the new check dams in the future.

**Fig. 7:**
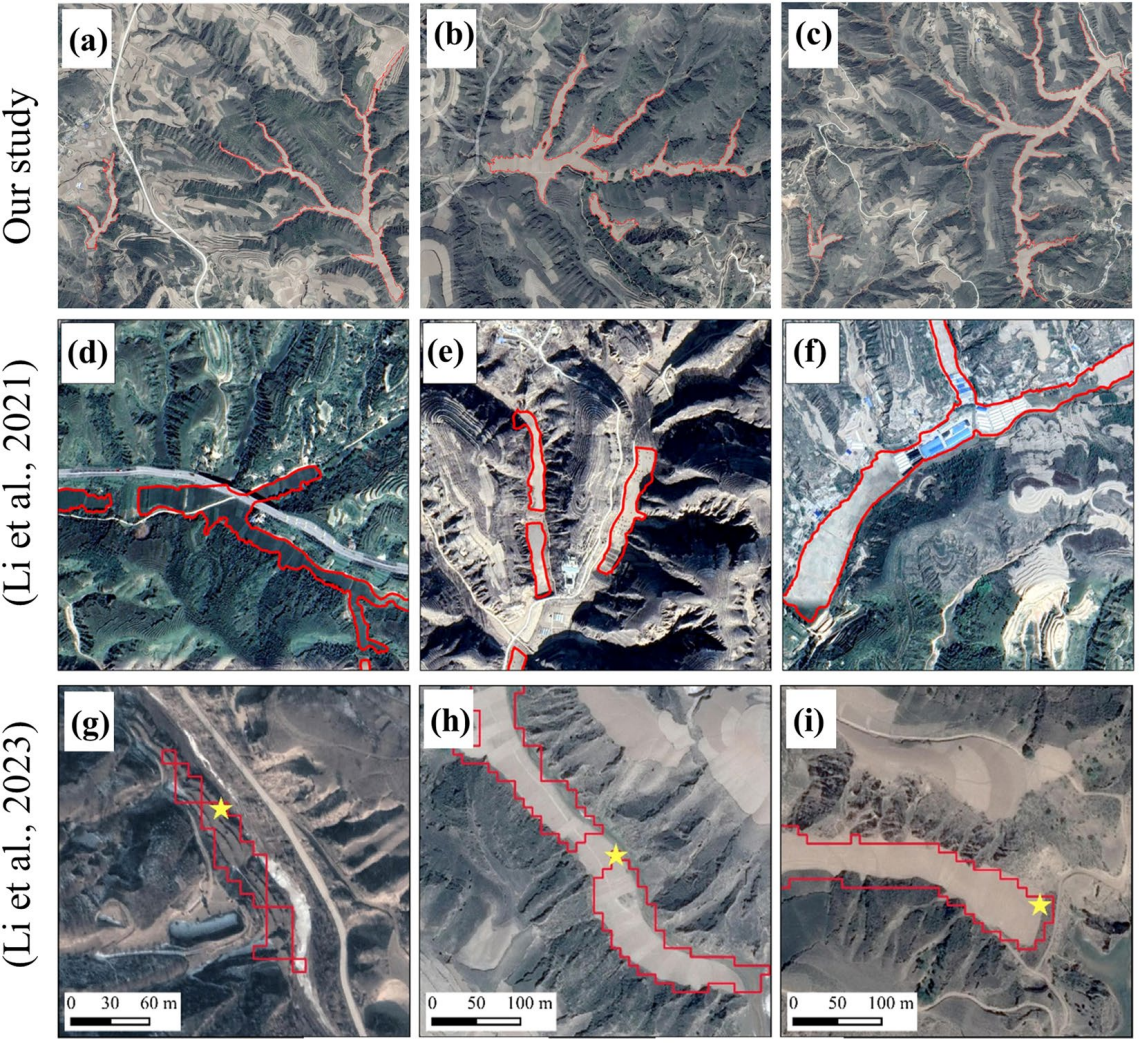
Comparison of our dam lands identification performance with previous studies. (**a**–**c**) Our study;(**d**–**f**) Dam lands extraction results based on deep learning (Li *et al*., 2021); (**g**–**i**) Dam lands extraction results based on ensemble learning models (Li *et al*., 2023).

## Data Availability

Multiresolution segmentation and assigned class algorithm are executed in the eCognition Developer 9.0. River network superposition and spatial statistical analysis are completed in ArcGIS 10.5. The calculation of NDVI time series is completed in Google Earth Engine (https://code.earthengine.google.com/d6f93ac6e504ec71de8607e746ebb84e).
